# Comparative Analysis of Amino Acid, Sugar, Acid and Volatile Compounds in 4-CPA-Treated and Oscillator-Pollinated Cherry Tomato Fruits During Ripe Stage

**DOI:** 10.3390/foods14223914

**Published:** 2025-11-15

**Authors:** Zhimiao Li, Sihui Guan, Meiying Ruan, Zhuping Yao, Chenxu Liu, Hongjian Wan, Qingjing Ye, Yuan Cheng, Rongqing Wang

**Affiliations:** 1 State Key Laboratory for Quality and Safety of Agro-Products, Vegetable Research Institute, Zhejiang Academy of Agricultural Sciences, Hangzhou 310021, China; 2 College of Agriculture, Shihezi University, Shihezi 832003, China; 3 Xianghu Laboratory, Hangzhou 311231, China

**Keywords:** 4-CPA, cherry tomato, aroma, amino acid, sugar, volatile compounds (VOCs), GC-MS, HPLC

## Abstract

4-Chlorophenoxyacetic acid (4-CPA) is an auxin-type plant growth regulator widely used in fruit and vegetable production. However, its influence on the nutritional and sensory qualities of horticultural crops remains insufficiently characterized. This study investigated the influence of 4-CPA application and oscillator-mediated pollination on the metabolic composition of fully ripe fruits of *Solanum lycopersicum* var. *cerasiforme* cv. ‘Zheyingfen No. 1’. Two concentrations of 4-CPA (16 mg/L and 8 mg/L) were applied during flowering, and their effects on amino acids, soluble sugars, organic acids, and volatile compounds (VOCs) were comparatively analyzed. The results indicated that treatment with 8 mg/L 4-CPA treatment significantly increased the total amino acid content in ripe fruits compared with the control and the 16 mg/L treatment. Among the 17 amino acids identified, the contents of umami-related amino acids, including glutamic acid (Glu) and aspartic acid (Asp), were markedly enhanced. In particular, Glu content in the C8 treatment was the highest and accounted for more than 50% of the total amino acid content. The accumulation of sugars was not significantly affected by 4-CPA treatment, while the C8 treatment resulted in the lowest level of total organic acids, which are crucial for flavor development at the ripening stage. A 29.35% increase in VOCs was observed” for conciseness in 4-CPA-treated fruits compared with the control. Analysis of relative odor activity values (rOAVs) showed that although 4-CPA treatment reduced the number of aroma-active compounds, it promoted the accumulation of β-ionone, thereby shifting the tomato fruit aroma profile toward floral, woody, sweet, and fruity notes. In summary, 4-CPA treatment regulated the nutritional and flavor quality of ripe cherry tomato fruits by increasing the content of Glu and other amino acids, enhancing the diversity of VOCs, and promoting the formation of key aroma-active substances such as β-ionone.

## 1. Introduction

Tomato (*Solanum lycopersicum* L.) is recognized as one of the most important horticultural crops worldwide. It originated in the Andes region of South America and was domesticated from wild relatives through prolonged selection and cultivation. Today, it is grown extensively across diverse agroecological zones and serves as a major source of vegetables for both fresh consumption and processing industries [1,2]. Tomato fruits are enriched with amino acids, soluble sugars, and organic acids, volatile compounds (VOCs) that not only contribute to nutritional value and human health but also play central roles in defining fruit flavor and sensory attributes [3]. Fruit quality is commonly assessed based on external characteristics such as size, shape, and coloration, as well as internal components that determine nutritional and organoleptic properties. Among internal quality parameters, amino acids, sugars, organic acids, and VOCs are regarded as key determinants of both nutritional content and consumer perception [4,5,6]. These metabolites act in concert to shape the flavor profile of tomato. Amino acids function not only as building blocks of proteins but also as biosynthetic precursors of flavor-active VOCs. Sugars and organic acids contribute to sweetness and acidity, thereby influencing taste balance. VOCs serve as the principal constituents of aroma, and even slight alterations in their concentration or composition can markedly affect sensory evaluation [7,8,9]. As consumer demand continues to shift toward tomatoes with improved flavor and nutritional quality, advancing fruit quality through precise and evidence-based strategies has become a major focus in contemporary plant biology and horticultural science.

The development of tomato fruit quality is a complex physiological and biochemical process influenced by genetic background, environmental conditions, and cultivation practices [10,11]. Among cultivation strategies, the application of plant growth regulators has been increasingly adopted in horticultural production to enhance fruit set, promote growth, and regulate developmental processes [12,13,14]. 4-Chlorophenoxyacetic acid (4-CPA), a synthetic auxin analog, is widely used in fruit and vegetable crops to promote parthenocarpic fruit development and improve yield, especially under conditions of limited pollination or environmental stress [15,16,17]. Extensive evidence has shown that 4-CPA can significantly increase fruit set and productivity in a variety of horticultural crops, including melon, sweet pepper, cherry, and pepino [13,16,18,19,20]. However, its physiological effects are highly dose dependent. While moderate concentrations promote fruit development, excessive application may lead to undesirable outcomes such as fruit deformation, reduced fruit size, and compromised appearance. Despite its broad agricultural use, most studies have focused on the external characteristics of 4-CPA-treated fruits, such as yield and shape. In contrast, its effects on internal quality traits, particularly those related to flavor and nutritional value, remain poorly understood. Compounds such as amino acids, soluble sugars, organic acids, and VOCs are fundamental determinants of tomato fruit quality [21,22]. However, little information is available on how 4-CPA influences the accumulation and composition of these key metabolites. Addressing this gap is essential for evaluating the comprehensive impact of 4-CPA on fruit quality and for optimizing its application in tomato cultivation.

In this study, the effects of 4-CPA application at two concentrations (16 mg/L and 8 mg/L) were systematically compared with oscillator-assisted pollination (CK) to evaluate how different 4-CPA treatments influence the quality attributes of mature cherry tomato fruits. Special emphasis was placed on key flavor- and nutrition-related metabolites, including amino acids, soluble sugars, organic acids, and VOCs. Unlike previous studies that mainly focused on yield or single metabolite groups, this research integrates amino acid metabolism and volatile compound profiling to comprehensively elucidate how 4-CPA modulates flavor formation at the biochemical level. The findings from this comparison are expected to provide a scientific basis for regulating fruit quality in tomato production and offer theoretical support for the rational use of plant growth regulators in horticultural crops.

## 2. Materials and Methods

### 2.1. Plant Material

Cherry tomato (*Solanum lycopersicum var. cerasiforme*) cv. ‘Zheyingfen No. 1’ was cultivated in spring 2024 at the Yangdu experimental greenhouse station of the Zhejiang Academy of Agricultural Sciences, Zhejiang, China (30°27′ N, 120°02′ E). Plants were managed under standard horticultural practices at a spacing of 40 cm within rows and 50 cm between rows.

Three fruit-setting treatments were applied to inflorescences using two pollination methods at anthesis (second to third trusses):

(i) Oscillator-assisted natural pollination (CK)—inflorescences were vibrated with a handheld oscillator once daily for three consecutive days between 09:00 and 11:00;
(ii) 4-CPA spray at 16 mg/L (C16)—flowers were sprayed once with a fine-mist hand sprayer, directing the spray to the abaxial side of the flowers and targeting the pedicel/calyx;
(iii) 4-CPA spray at 8 mg/L (C8)—identical to C16 except for concentration.

Treated inflorescences were labelled with the application date. For each treatment, 12 plants were included and grouped into three independent biological replicates (four plants per replicate). Fruits were harvested 45 days after treatment (D45), corresponding to the full-ripe stage, from the labelled trusses only. Within each biological replicate, fruits collected from the four plants were pooled to constitute the replicate sample for downstream analyses; the pooled mass and assay-specific aliquots are reported in the subsequent methods. Only sound fruits without visible defects were used.

### 2.2. Determination of Total Amino Acids (AA) and Individual Amino Acid Profiles

Amino acids were extracted from approximately 0.2 g of homogenized tomato samples using 1.5 mL of 70% ethanol, followed by ultrasonic extraction for 30 min and centrifugation at 12,000 rpm for 10 min. The supernatant was evaporated to dryness under nitrogen and re-dissolved in 1 mL of ultrapure water. Prior to HPLC analysis, amino acids were derivatized with phenylisothiocyanate (PITC) in the presence of triethylamine for 1 h at room temperature. Chromatographic separation was performed using a Wufeng LC-100 HPLC system equipped with a dedicated amino acid analysis column (250 mm × 4.6 mm, 5 µm). The mobile phases consisted of 0.05 mol L^−1^ sodium acetate buffer (pH 6.5) (A) and methanol: acetonitrile: water = 20:60:20 (*v*/*v*/*v*) (B) with a flow rate of 1.0 mL min^−1^. Detection was carried out at 254 nm using a UV detector. Seventeen amino acids were quantified using external standards, and calibration curves (R^2^ > 0.999) were established for each amino acid to ensure quantitative accuracy.

### 2.3. Determination of Soluble Sugars and Organic Acids

Soluble sugars, including glucose, fructose, and sucrose, were quantified using an LC-100 high-performance liquid chromatography (HPLC) system equipped with a Dikma Polyamino HILIC column (250 mm × 4.6 mm, 5 µm). Approximately 0.2 g of each homogenized sample was extracted with 1 mL of 40% acetonitrile aqueous solution by grinding, followed by ultrasonic extraction at room temperature for 30 min. The extract was then centrifuged at 12,000 rpm for 10 min at room temperature. The supernatant was filtered through a syringe filter and subjected to HPLC analysis. Chromatographic separation was performed under isocratic conditions using acetonitrile (A) and water (B) at a ratio of 60:40 (*v*/*v*) as the mobile phase, with a flow rate of 1.0 mL min^−1^ and a column temperature of 40 °C. Detection was carried out with a refractive index (RI) detector (Shodex RI-201H, Showa Denko K.K., Tokyo, Japan), and the injection volume was 10 µL.

Organic acids, including citric acid and malic acid, were determined using a Shimadzu LC-20AT HPLC system (Shimadzu Corporation, Kyoto, Japan) equipped with a C18 reverse-phase column (150 mm × 4.6 mm, 5 µm). A total of 0.2 g of each sample was extracted with 1 mL of ultrapure water after thorough homogenization. The mixture was ultrasonicated for 30 min, followed by centrifugation at 12,000 rpm for 10 min at 4 °C. The resulting supernatant was filtered through a syringe filter and analyzed by HPLC. The mobile phase consisted of methanol (A) and 0.2% sodium dihydrogen phosphate aqueous solution (pH 2.7) (B) mixed at a ratio of 3:97 (*v*/*v*). The flow rate was 0.6 mL min^−1^, the column temperature was maintained at 30 °C, and detection was performed at 210 nm using a UV detector (SPD-20A, Shimadzu Corporation, Kyoto, Japan). The injection volume was 10 µL.

### 2.4. Identification of VOCs by GC–MS

After harvesting, tomato fruits were immediately weighed, flash-frozen in liquid nitrogen, and stored at −80 °C until further analysis. The frozen samples were ground into a fine powder under liquid nitrogen. A total of 500 mg of powdered tissue was transferred into a 20 mL headspace vial (Agilent, Palo Alto, CA, USA), to which 10 µL of internal standard solution (3-Hexanone-2,2,4,4-d_4_, 10 µg mL^−1^ in methanol; CDN Isotopes Inc., Pointe-Claire, QC, Canada; CAS No. 24588-54-3) was added, followed by the addition of saturated sodium chloride solution to inhibit enzymatic activity. The vial was sealed with a TFE-silicone septum screw cap (Agilent). For headspace solid-phase microextraction combined (HS-SPME), the vials were incubated at 60 °C for 5 min to equilibrate, and then a 120 µm DVB/CWR/PDMS fiber (Agilent) was exposed to the headspace for 15 min at the same temperature. Each extraction was performed in triplicate to ensure reproducibility. Calibration was conducted using selected reference standards to ensure accurate compound identification and quantification. Desorption of VOCs from the fiber was performed in the injector of a gas chromatograph (model 8890, Agilent) at 250 °C for 5 min in splitless mode. VOCs were separated and identified using a gas chromatography–mass spectrometry (GC–MS) system comprising an Agilent 8890 gas chromatograph and a 7000E mass spectrometer (Agilent), equipped with a DB-5MS capillary column (30 m × 0.25 mm × 0.25 µm, 5% phenyl-polymethylsiloxane). Helium was used as the carrier gas at a constant linear velocity of 1.2 mL/min. The injector temperature was maintained at 250 °C. The oven temperature program was as follows: initial temperature of 40 °C held for 3.5 min, ramped at 10 °C/min to 100 °C, then at 7 °C/min to 180 °C, followed by 25 °C/min to 280 °C and held for 5 min. Mass spectra were acquired under electron impact (EI) ionization at 70 eV. The temperatures of the quadrupole, ion source, and transfer line were set to 150 °C, 230 °C, and 280 °C, respectively. VOCs were identified and quantified using the selected ion monitoring (SIM) mode.

### 2.5. Qualitative and Quantitative Analysis of Metabolites

Qualitative and quantitative analyses of metabolites were performed based on a self-established database developed by Metware Biotechnology Co., Ltd. (Wuhan, China). The database was constructed using information from multiple species, published literature, authentic standards, and retention indices (RIs). Each compound was monitored in selected ion monitoring (SIM) mode with one quantitative ion and two to three qualitative ions. All target ions were detected sequentially according to their retention time windows. A compound was considered positively identified when the retention time matched that of the reference standard and the selected characteristic ions were simultaneously detected in the background-subtracted sample mass spectrum [23]. Quantification was achieved by integrating the peak area of the quantitative ion, which enhanced the accuracy and reproducibility of metabolite quantification.

### 2.6. rOAV Analysis

The relative odor activity value (rOAV) is a threshold-based approach used to identify key aroma-active compounds in horticultural crops [24]. It reflects the contribution of each VOC to the overall aroma profile of a sample. In this study, the compound with the highest rOAV in tomato fruits was set to 100 as the reference. Compounds with rOAV values ≥ 1 were considered key aroma contributors, while those with 0.1 < rOAV < 1 were classified as aroma modifiers [25]. Odor thresholds (Tᵢ) for each compound were obtained from previously published literature rather than experimentally determined. rOAVᵢ = Cᵢ/Tᵢ, where rOAVᵢ represents the relative odor activity value of compound i, Cᵢ is the relative concentration of the compound (µg/g or µg/mL), and Tᵢ is the odor threshold of the compound (µg/g or µg/mL) [26,27].

### 2.7. Statistical Analysis

Data processing and compilation were performed using Excel 2021 (Microsoft Corporation, Redmond, WA, USA). Statistical analyses and bar graph visualizations were conducted using GraphPad Prism 10 (GraphPad Software, San Diego, CA, USA). One-way analysis of variance (ANOVA) was performed using IBM SPSS Statistics version 26 (IBM Corporation, Armonk, NY, USA) to evaluate the significance of differences among treatments. Heatmaps, Venn diagrams, and related visualizations were generated using the online platform available at https://cloud.metware.cn. Post hoc comparisons were performed using the least significant difference (LSD) test at a significance level of *p* < 0.05.

## 3. Results and Discussion

### 3.1. The Effect of 4-CPA Treatment on the Soluble Sugar and Organic Acid Contents of Tomato Fruits at the Ripe Stage

**Figure 1:**
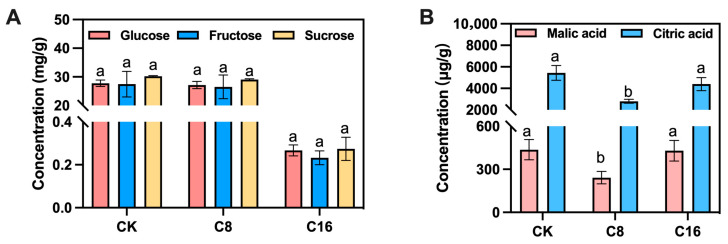
Sugar and acid contents in cherry tomato fruits treated with 4-CPA and natural pollination. (**A**) soluble sugar; (**B**) organic acid. CK, C8, and C16 represent the cherry tomato fruits 45 days after natural pollination, treatment with half the standard concentration, and conventional concentration 4-CPA spray treatment, respectively (mature stage). Different letters indicate significant differences between treatments. *p* < 0.05.

Sugars and acids are the major determinants of the taste and quality of cherry tomato fruit [28]. HPLC analysis was performed to compare the accumulation of soluble sugars (glucose, fructose, and sucrose) and acids (malic acid and citric acid) in tomato ripe stage of oscillator-assisted natural pollination and 4-CPA treatment. The sucrose content in ripe fruits was relatively low, accounting for only about 1% of fructose or glucose, and no significant differences were observed among the different 4-CPA concentrations and the control (Figure 1A). This result is consistent with the finding of Nandwani et al. [29]. As an auxin analogue, 4-CPA primarily promotes cell division and expansion rather than directly regulating sugar metabolism–related enzymes such as sucrose synthase and soluble acid invertase. Furthermore, because 4-CPA–induced fruit development bypasses normal fertilization, it may lack the hormonal cascade (e.g., ethylene and ABA) that activates sugar metabolism pathways, thereby limiting sugar accumulation [30,31]. Meanwhile, enhanced fruit set caused by 4-CPA may increase sink demand without a proportional rise in photosynthetic carbon supply, leading to a source–sink imbalance and a dilution effect on fruit sugar concentration [32]. Consequently, the effect of 4-CPA on soluble sugar content was not significant, likely due to the combined influences of auxin-mediated hormonal regulation and source–sink redistribution.

**Figure 2:**
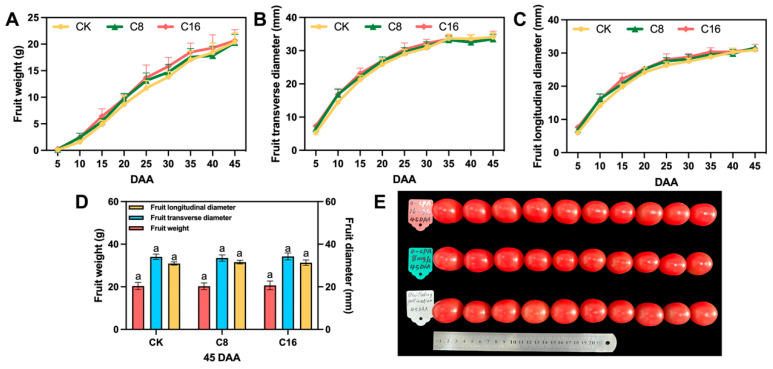
Effects of 4-CPA treatment and natural pollination on the appearance quality of tomato fruits. (**A**) Single fruit weight of different developmental tomatoes; (**B**) Fruit transverse diameter of different developmental tomatoes; (**C**) Fruit longitudinal diameter of different developmental tomatoes; (**D**) The physical characteristics of the fruits under the three treatments at 45DAA; (**E**) Fruit phenotypic diagrams under the three treatments at 45DAA. DAA, day after anthesis. Values are expressed as means ± SE of at least 10 replicates. Different letters indicate significant differences between treatments. *p* < 0.05.

In addition to sugar content, organic acids are key contributors to the flavor profile of cherry tomato fruits. In the present study, both malic acid and citric acid contents were significantly lower in fruits treated with C8 at the ripe stage, compared with those in the CK and C16 treatments (Figure 1B). Malic acid accounted for only about one-thirteenth of the citric acid content, confirming citric acid as the predominant organic acid and main contributor to perceived acidity [33,34]. Exogenous application of 4-CPA led to a reduction in total organic acid levels, particularly in citric acid, with the most pronounced decrease observed under the C8 treatment. Given that citric acid directly influences perceived acidity and the sugar–acid balance, its decline may substantially impact flavor quality. The moderate accumulation of both sugars and acids in ripe fruits is known to enhance palatability and consumer acceptance [35]. These results suggest that the C8 treatment may contribute to a more favorable flavor profile in fully ripe cherry tomato fruits. Furthermore, the fruit weight and size were also assessed at maturity. No significant differences were observed among the different treatments, indicating that 4-CPA application did not affect the physical traits of mature cherry tomato fruits (Figure 2).

### 3.2. Change in VOCs in Cherry Tomato Ripe Stage

**Figure 3:**
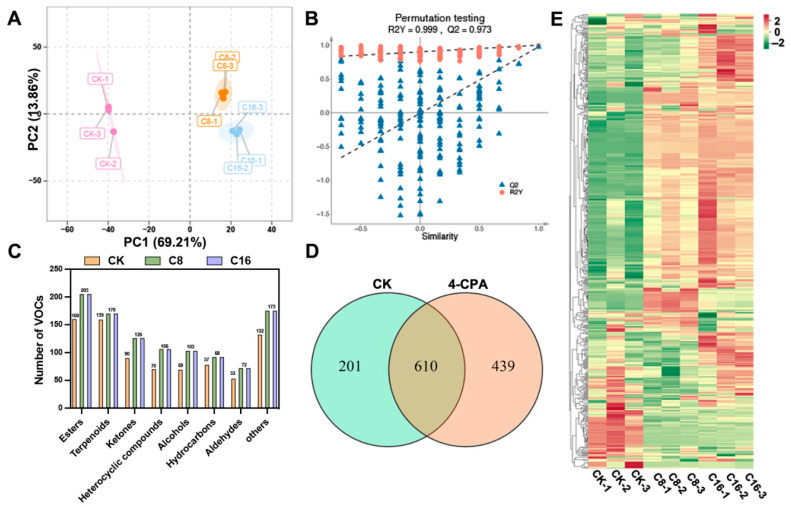
Multivariate and compositional analysis of VOCs in cherry tomato fruits under different treatments. (**A**) PCA score plot showing sample separation among CK, C8 and C16; (**B**) OPLS-DA permutation test confirming model reliability; (**C**) Distribution of VOC categories detected by HS-SPME–GC–MS; (**D**) Venn diagram showing shared and unique VOCs across treatments; (**E**) Heatmap of 610 shared VOCs illustrating relative abundance patterns among groups.

VOCs, together with sugars and organic acids, are key determinants of fruit flavor and exert substantial influence on consumer preference. To explore the differences in VOC profiles among cherry tomato fruits subjected to different 4-CPA treatments, principal component analysis (PCA) was first performed (Figure 3A). As an unsupervised method for multivariate data analysis, PCA enables pattern recognition by reducing data dimensionality while retaining the majority of variance [36]. In this case, the first two principal components (PC1 and PC2) explained 69.21% and 13.86% of the total variance, respectively, cumulatively accounting for over 83% of the total variability. The PCA score plot revealed clear separation among the CK, C8, and C16 groups, indicating substantial differences in VOC composition across treatments. To further enhance group discrimination, orthogonal partial least squares discriminant analysis (OPLS-DA) was conducted [37]. To assess the robustness and reliability of the OPLS-DA model, permutation testing was performed (Figure 3B). The model exhibited excellent performance, with R^2^Y = 0.999 and Q^2^ = 0.973, both significantly higher than those obtained from permuted models. Furthermore, the Q^2^ regression line displayed a pronounced negative slope, indicating that the model was not overfitted. These results collectively suggest that the OPLS-DA model effectively captured group-specific variations in aroma profiles and provided strong predictive power.

In this study, HS–SPME-GC–MS was employed to characterize the volatile profiles and quantify the total volatile content in cherry tomato fruits subjected to CK and two concentrations of 4-CPA treatment during the ripe stage. A total of 1250 VOCs were identified across all samples, encompassing esters (242, 19.36%), terpenoids (226, 18.08%), ketones (147, 11.76%), alcohols (124, 9.92%), heterocyclic compounds (119, 9.52%), hydrocarbons (112, 8.96%), aldehydes (80, 6.40%), and other VOCs including acids (58, 4.64%), phenols (47, 3.76%), amines (42, 3.36%), ethers (26, 2.08%), nitrogen-containing compounds (17, 1.36%), halogenated hydrocarbons (6, 0.48%), and sulfur-containing compounds (4, 0.32%) (Table S1). Notably, 4-CPA application markedly altered the volatile composition. The CK group exhibited 811 VOCs, whereas both C8 and C16 treatments yielded 1049 VOCs, corresponding to a 29.35% increase compared with natural pollination. As shown in Figure 3C, the number of compounds across nearly all volatile categories increased following 4-CPA application. Venn diagram analysis (Figure 3D) revealed that 610 volatiles were common among the treatment groups, including 123 esters, 103 terpenoids, 69 ketones, 58 hydrocarbons, 57 heterocyclic compounds, 48 alcohols, 45 aldehydes, 34 acids, 30 phenols, 13 ethers, 11 amines, 10 nitrogen-containing compounds, 5 halogenated hydrocarbons, and 4 sulfur-containing compounds. Among these, esters emerged as the predominant contributors to the shared aroma profile. Furthermore, heatmap visualization of the shared volatiles (Figure 3E) revealed a general upregulation of volatile contents in 4-CPA-treated fruits, with the C16 group showing the highest intensities across most compounds.

**Figure 4:**
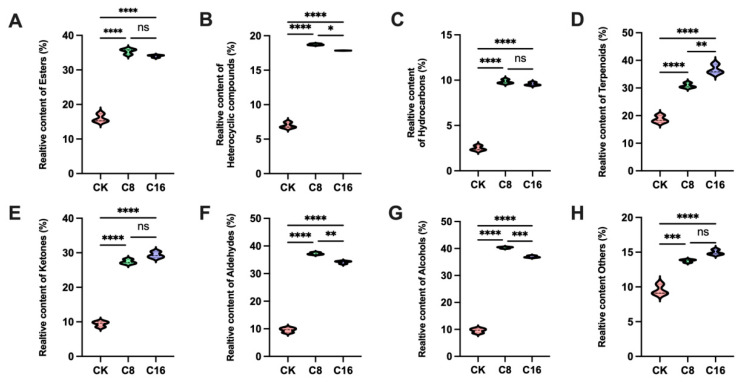
Relative content of main (**A**) esters; (**B**) heterocyclic compounds; (**C**) hydrocarbons; (**D**) terpenoids; (**E**) ketones; (**F**) aldehydes; (**G**) alcohols and (**H**) others in cherry tomato at different treatments at the ripe stage. Bars show mean ± SD; significance is indicated as  *p* < 0.05 (*), *p* < 0.01 (**), *p* < 0.001 (***), *p* < 0.0001 (****); ns, not significant.

The relative contents of total and major VOCs in fruits at the ripe stage were further analyzed. These findings suggest that 4-CPA treatment significantly enhanced the accumulation of most volatile categories, with a dose-dependent pattern observed for terpenoids, ketones, alcohols, and others. Notably, the contents of heterocyclic compounds, aldehydes and alcohols peaked under the C8 treatment but declined slightly at C16, indicating potential inhibition of their biosynthesis at higher concentrations (Figure 4B,F,G). In contrast, esters, hydrocarbons and ketones were significantly elevated in both C8 and C16 treatments compared to CK, but showed no significant differences between the two concentrations (Figure 4A,C,E). This indicates that while 4-CPA strongly induces ester and hydrocarbon accumulation, their biosynthesis may be less responsive to concentration gradients, possibly due to metabolic saturation or feedback regulation in downstream pathways. Together, these results demonstrate that 4-CPA broadly reshapes the tomato volatile profile, though the extent and pattern of induction vary by compound class.

### 3.3. The Effect of 4-CPA Treatment on the Amino Acid Contents of Tomato Fruits at the Ripe Stage

**Figure 5:**
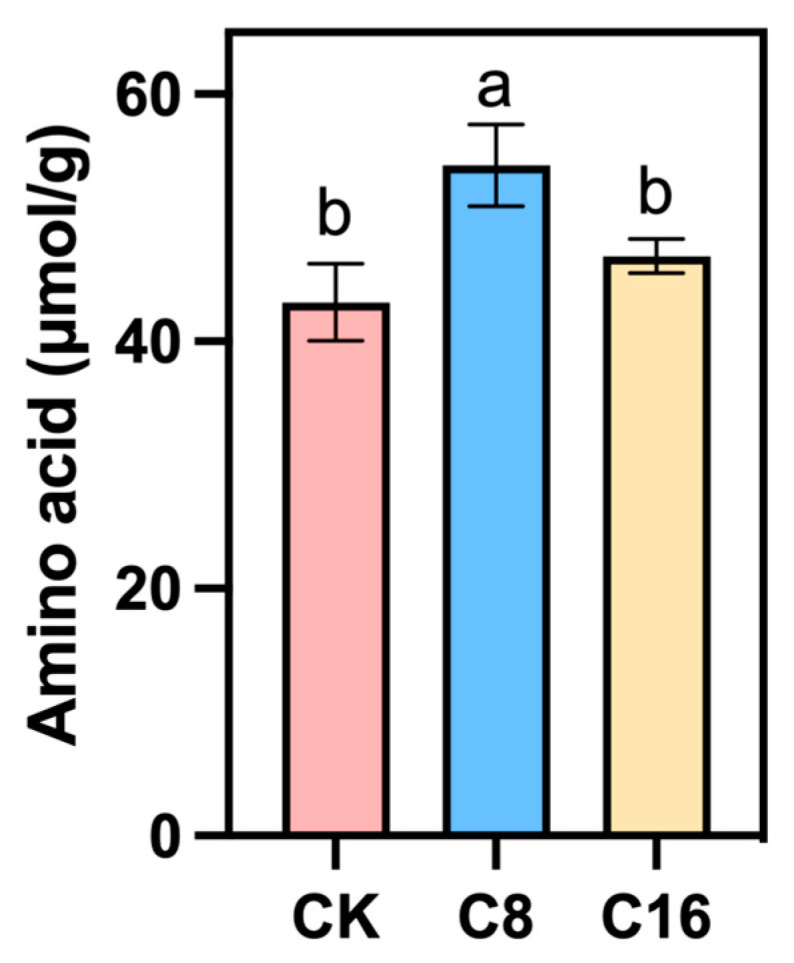
Content of total AA in cherry tomato fruits treated with 4-CPA and those under natural pollination. Different letters indicate significant differences between treatments. *p* < 0.05.

Amino acids (AA) are an important nutritional component in cherry tomato fruits, not only influencing the flavor of the fruits, but also being of great significance to the nutritional value of the fruits [38]. The experimental results show that in the fully ripe fruits, the total AA content of the fruits treated with two concentrations of 4-CPA was higher than that of CK, and the total AA of the tomatoes treated with C8 was the highest, indicating that the treatment with 4-CPA is conducive to the accumulation of total AA in the cherry tomato fruits during the ripening stage (Figure 5). Although no previous studies have directly reported that 4-CPA enhances amino acid accumulation in tomato fruits, several investigations have demonstrated that auxin and auxin-like compounds can modulate amino acid metabolism during fruit development and ripening [35]. Therefore, the increase in total amino acid content observed under 4-CPA treatment in this study may be partly attributed to its auxin-like regulatory effects.

In order to further explore the effects of 4-CPA treatment on the content of different types of amino acids in tomato fruits, this study used HPLC to detect and compare the contents of 17 amino acids in cherry tomato fruits treated with different concentrations of 4-CPA. The results showed that the contents of Asp and Glu in the fruits treated with different concentrations of 4-CPA were higher than those in the CK, especially in the C8-treated fruits, where the differences were significant compared to the control (Table 1). In contrast, the contents of 9 amino acids such as Thr, Val, Tyr, Ile, Leu, Arg, Cys, Lys and Ala in the control fruits were higher than those in the fruits treated with different concentrations of 4-CPA. The contents of these amino acids in the C16-treated tomato fruits were the lowest (Table 1). For other amino acids, such as His, Pro, Met, Ser, Gly and Phe, there were no significant differences in their contents between the 4-CPA-treated fruits and the natural pollination fruits. It is worth noting that the total content of these 17 amino acids in the 4-CPA-treated fruits was higher than that in CK, especially in the C8 fruits, where the total content of 17 amino acids was the highest (Table 1), and its change trend was the same as the experimental results of total amino acid content determined by the ninhydrin colorimetric method used in this study. These results suggest that 4-CPA may differentially modulate amino acid metabolism pathways, promoting the accumulation of umami-related amino acids such as Glu and Asp while suppressing the synthesis of certain branched-chain and aromatic amino acids. Similar trends have been observed under auxin or hormonal regulation in tomato and other fruit crops, where specific amino acid biosynthetic routes are preferentially activated or repressed in response to exogenous signals [39].

AA not only serve as building blocks for protein biosynthesis but also play a crucial role as precursors in the formation of volatile aroma compounds in fruits. In tomato, several key volatiles such as aldehydes, alcohols, and esters are known to originate from the catabolism of branched-chain amino acids (valine, leucine, isoleucine) and aromatic amino acids (phenylalanine, tyrosine) through transamination and decarboxylation pathways [40]. For instance, L-phenylalanine can be converted into floral-scented volatiles like 2-phenylethanol and phenylacetaldehyde, while branched-chain amino acids contribute to fruity and malty volatiles such as 3-methylbutanol and 2-methylbutanal [41]. In this study, distinct changes were observed in the levels of aroma-related amino acids under different concentrations of 4-CPA treatment. In C8-treated fruits, the levels of Tyr and Cys were significantly reduced compared to CK. Tyrosine is a known precursor for phenolic volatiles, while cysteine participates in sulfur-containing volatile formation, both of which are important contributors to tomato’s characteristic aroma complexity [42]. The decreased abundance of these two amino acids under C8 treatment may negatively affect the generation of their corresponding volatiles. More notably, in the C16-treated fruits, a broader set of amino acids exhibited significant reductions, including Thr, Val, Tyr, Ile, Leu, Arg, Lys, and Ala. Among these, Val, Ile, and Leu are key branched-chain amino acids associated with the biosynthesis of fruity and malty volatiles, while Ala and Thr also serve as substrates for aldehyde and alcohol production [5]. The widespread reduction in these amino acids in C16-treated fruits suggests a more substantial impairment in the formation of VOCs, which may result in a more pronounced decline in fruit aroma and flavor perception. Taken together, while 4-CPA treatment enhanced the overall amino acid content, especially the accumulation of umami-related amino acids such as Asp and Glu, it simultaneously suppressed the levels of several key volatile precursors in a concentration-dependent manner. These changes are likely to influence the balance between taste-active and aroma-active components, potentially altering the sensory profile of cherry tomato fruits. Further investigation at the metabolic and transcriptomic levels is warranted to elucidate the regulatory mechanisms underlying this shift in amino acid–aroma linkage.

### 3.4. The Effect of 4-CPA Treatment on the Key Volatiles of Tomato Fruits at the Ripe Stage

The impact of VOCs on the overall aroma characteristics of tomatoes depends not only on the types and concentrations of VOCs, but also on their odor thresholds. Certain volatiles, despite being present at low concentrations, may exert a significant influence on the overall aroma due to their low odor thresholds. The rOAV is a widely used metric to assess the aroma contribution of individual VOCs. An rOAV ≥ 1 indicates that the compound has a direct impact on the fruit’s overall aroma, while compounds with 0.1 < rOAV < 1 are considered to have a modifying or enhancing effect [43]. For each treatment, rOAVs were scaled relative to the highest-impact compound (set to 100). Based on the relative odor activity value (rOAV ≥ 1), a total of 12 key VOCs were identified across the all groups (Table 2). These volatiles include ketones, terpenoids, esters, aldehydes, and sulfur compounds, each contributing distinctive odor characteristics to tomato aroma. The number of key aroma-active compounds was highest in the CK (8 compounds) and decreased with 4-CPA application: 7 in C8 and only 6 in C16. This indicates that 4-CPA treatment may lead to a loss in aroma complexity by reducing the diversity of dominant odor-contributing volatiles. Among all compounds, (5Z)-octa-1,5-dien-3-one, a ketone with geranium-like and metallic odor notes, exhibited the highest rOAV (100.00) in CK and remained abundant in C8 and C16, albeit at slightly reduced levels. In contrast, β-Ionone, a floral-woody terpenoid derived from carotenoid cleavage, showed a striking increase under 4-CPA treatment, with rOAV values of 100.00 in both C8 and C16, compared to 51.50 in the control. This suggests that 4-CPA application may enhance β-ionone biosynthesis, thereby shifting the aroma profile toward a more floral and sweet character. Previous studies have confirmed that β-ionone is one of the most impactful volatiles contributing to tomato flavor and consumer preference [5,44]. Its low odor threshold and distinct sensory characteristics make it a key indicator for flavor quality assessment in tomato breeding and postharvest studies.

Other important volatiles such as 1-octen-3-one (mushroom odor) and trans-β-ionone (floral, woody) also emerged as contributors under 4-CPA treatment but were absent or negligible in the control. For example, 1-octen-3-one showed rOAVs of 20.82 and 16.57 in C8 and C16, respectively, while trans-β-ionone maintained a moderate presence across all treatments. Notably, 3-mercapto-3-methylbutylformate, a sulfur-containing ester with onion-like aroma, was detected only in the 4-CPA groups (rOAV = 2.15 in C8 and 1.14 in C16), indicating that 4-CPA may induce sulfur-containing compound biosynthesis. On the other hand, several aldehydes (e.g., (E)-2-nonenal, (E,Z)-2,6-nonadienal, hexanal) which contribute green and cucumber-like notes, were only active (rOAV ≥ 1) in the control or at lower levels under C8 treatment, but fell below odor threshold under C16, suggesting that high-concentration 4-CPA may suppress lipid-derived aldehyde production. Taken together, these results suggest that 4-CPA treatment modifies the volatile landscape of cherry tomato fruits in a concentration-dependent manner. While it enhances compounds like β-ionone that are strongly odor-active even at low concentrations, it concurrently reduces the number and diversity of other key volatiles, potentially leading to a more simplified or more intense unique flavor profile at full ripening. Such compound-specific alterations are likely mediated through the differential regulation of precursor pathways, including carotenoid cleavage for terpenoids and lipid oxidation for aldehydes, both of which are known to be modulated by auxin-responsive signals [45]. The induction of sulfur-containing volatiles also suggests that 4-CPA may affect methionine or cysteine-derived aroma pathways, as previously observed in melon and tomato [46].

**Figure 6:**
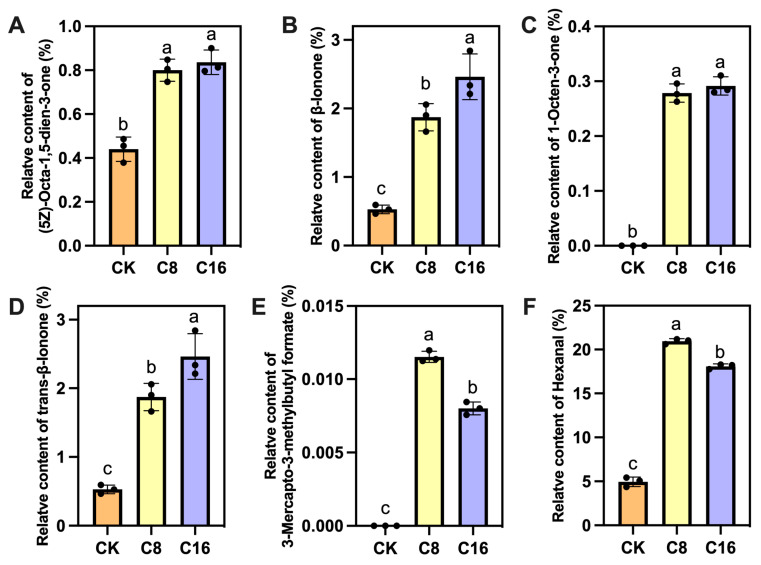
Relative contents of 6 key VOCs (rOAV ≥ 1) in fruits treated with different 4-CPA treatment. (**A**) (5Z)-Octa-1,5-dien-3-one; (**B**) β-Ionone; (**C**) 1-Octen-3-one; (**D**) trans-β-Ionone; (**E**) 3-Mercapto-3-methylbutylformate; (**F**) Hexanal. The black dots represent the values of each treatment. Bars with different letters differ at *p* < 0.05 (one-way ANOVA, LSD).

The relative contents of 6 key aroma-active compounds (rOAV ≥ 1) were analyzed under the C16 treatment (Figure 6). The results indicated that, except for 3-Mercapto-3-methylbutylformate (rOAV = 1.14, Figure 6E) and hexanal (rOAV = 1.03, Figure 6F), which exhibited the highest levels in the C8 treatment, the remaining four compounds showed their highest concentrations in the C16 treatment (Figure 6A–D). All 6 aroma-active compounds displayed significantly higher contents in both 4-CPA treatments compared with CK. Notably, β-ionone exhibited the highest rOAV value among the detected VOCs following 4-CPA application (rOAV = 100, Figure 6B), and its content was significantly greater in the C16 treatment than in C8. Furthermore, the concentrations of three additional compounds with relatively high rOAV values, (5Z)-octa-1,5-dien-3-one, 1-octen-3-one, and trans-β-ionone, were markedly elevated in the 4-CPA-treated fruits compared to the CK.

### 3.5. Alterations in Aroma Diversity and Sensory Composition of Cherry Tomato Induced by 4-CPA Treatment

**Figure 7:**
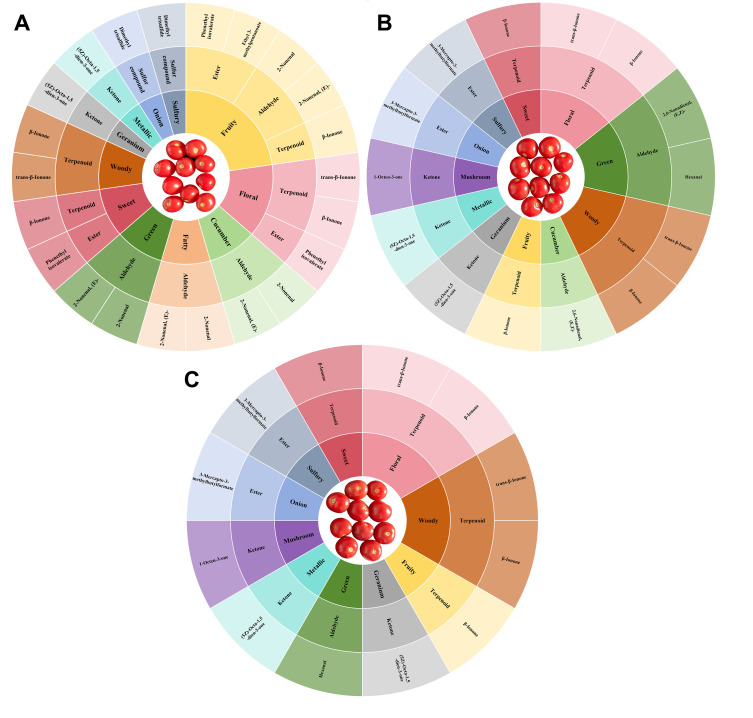
Sensory characteristics and flavor wheels of key VOCs for different samples. (**A**) CK flavor wheel; (**B**) C8 flavor wheel; (**C**) C16 flavor wheel.

To present the overall sensory flavor characteristics of tomato fruits in greater detail, a flavor wheel was constructed for all groups (Figure 7). This visualization highlights variations in aroma diversity and sensory composition among treatments. In terms of the number and type of sensory descriptors, the CK exhibited 11 distinct aroma notes, representing a relatively rich and balanced aroma profile. 4-CPA treatment altered the sensory flavor composition, leading to a simplified aroma profile dominated by specific notes. Although the C8 group also retained 11 sensory descriptors, it showed enhanced mushroom-like and reduced fatty aromas compared with CK. The C16 group displayed only 10 sensory descriptors, with the cucumber-like note absent, suggesting that higher concentrations of 4-CPA may suppress certain freshness-related aroma traits. Specifically, CK fruits contained 2 sweet, 2 green, 5 fruity, and 3 floral aroma-contributing compounds, whereas in 4-CPA-treated fruits, each sensory category was mainly driven by one or two representative VOCs. This indicates a decline in aroma diversity following 4-CPA application, especially under the C16 treatment. Previous studies have emphasized that aroma complexity and sensory harmony are closely tied to the richness and balance of key low-threshold volatiles, which shape consumer perception and acceptance of tomato flavor [45]. The simplification of sensory profiles observed here suggests that 4-CPA treatment disrupts the balance of low-threshold volatiles critical for maintaining aromatic complexity, shifting the flavor profile toward a more monotonous character. Notably, β-ionone, a floral, woody, sweet, and fruity compound with an extremely low odor threshold, became the dominant contributor in 4-CPA-treated fruits. This shift indicates that although 4-CPA promotes the accumulation of certain high-impact volatiles, it simultaneously reduces the diversity of compound classes, resulting in a simplified aroma structure. Similar findings have shown that reductions in green, fresh, and sulfurous volatiles, along with enrichment of apocarotenoid-derived compounds like β-ionone, can significantly alter sensory perception by amplifying only a few key aroma traits at the expense of overall complexity [5,47].

### 3.6. Toxicological and Ecological Considerations of 4-CPA Application

Although 4-CPA has been widely used as an auxin-type plant growth regulator in horticultural production, its toxicological and ecological implications warrant attention. Previous studies have shown that various chlorophenoxyacetate derivatives exhibited enhanced ecotoxicity toward aquatic organisms such as Vibrio fischeri, Daphnia magna, and Pimephales promelas, indicating potential non-target aquatic risk of 4-CPA analogs [48]. Acute toxicity studies in rice demonstrated that 4-CPA significantly inhibited root growth at concentrations between 2 mg/L and 10 mg/L, highlighting the possibility of unintended effects on non-crop plants [49]. From a horticultural perspective, exogenous spraying of 4-chlorophenoxyacetic acid sodium salt (4-CPANa) was shown to promote growth and flavonoid biosynthesis in mulberry leaves, underscoring its broad bioactivity beyond target effects [50]. Therefore, while the application of 4-CPA in tomato production may enhance yield and flavor traits, its use should be accompanied by careful assessment of residue levels, pre-harvest intervals, and weed and non-target-organism consequences to balance production benefits with environmental safety.

## 4. Conclusions

In this study, we comprehensively investigated the effects of 4-CPA treatment on the nutritional and flavor quality of cherry tomato fruits at the ripe stage. While sugar content remained relatively stable, the C8 treatment significantly reduced citric acid levels, potentially improving the sugar–acid balance and enhancing taste perception. The application of 4-CPA increased the diversity of VOCs by 29.35%, particularly elevating key floral and fruity VOCs such as β-ionone. Although the number of aroma-active compounds (rOAV ≥ 1) was slightly reduced, the accumulation of high-impact VOCs contributed to a more concentrated and intensified aroma profile. Moreover, the enhancement of umami-related amino acids, including Glu and Asp, further enriched the flavor. From a practical perspective, moderate 4-CPA application (around 8 mg/L) appears optimal for improving sweetness, aroma intensity, and flavor uniformity in commercial tomato cultivation without compromising fruit development. Future research should also examine other important quality parameters, such as antioxidant compounds (e.g., lycopene, vitamin C), postharvest shelf life, and sensory evaluation, to provide a more comprehensive understanding of how 4-CPA influences both the nutritional and consumer-perceived qualities of tomato fruits. These efforts will contribute to the optimized application of 4-CPA in cherry tomato cultivation and provide a scientific basis for its use in horticultural production.

## Figures and Tables

**Table 1:** Total amino acid contents of pollinated and 4-CPA treated cherry tomato fruits (mg/g. FW).

Amino Acid	CK	C8	C16
Aspartic acid (Asp)	2.171 ± 0.220 ^b^	2.715 ± 0.284 ^a^	2.390 ± 0.120 ^ab^
Glutamic acid (Glu)	11.619 ± 0.924 ^b^	16.748 ±3.742 ^a^	14.844 ± 0.974 ^ab^
Threonine (Thr)	5.090 ± 0.647 ^a^	4.516 ± 1.021 ^ab^	3.187 ± 0.111 ^b^
Valine (Val)	0.103 ± 0.004 ^a^	0.072 ± 0.029 ^ab^	0.051 ± 0.003 ^b^
Tyrosine (Tyr)	0.143 ± 0.003 ^a^	0.098 ± 0.035 ^b^	0.076 ± 0.003 ^b^
Isoleucine (Ile)	0.121 ± 0.003 ^a^	0.099 ± 0.021 ^ab^	0.076 ± 0.001 ^b^
Leucine (Leu)	0.217 ± 0.010 ^a^	0.176 ± 0.038 ^ab^	0.131 ± 0.002 ^b^
Arginine (Arg)	0.331 ± 0.028 ^a^	0.302 ± 0.006 ^a^	0.241 ± 0.004 ^b^
Cysteine (Cys)	0.127 ± 0.001 ^a^	0.118 ± 0.007 ^b^	0.121 ± 0.002 ^ab^
Lysine (Lys)	0.296 ± 0.018 ^a^	0.250 ± 0.053 ^ab^	0.213 ± 0.003 ^b^
Alanine (Ala)	0.479 ± 0.032 ^a^	0.433 ± 0.053 ^ab^	0.399 ± 0.025 ^b^
Histidine (His)	0.314 ± 0.020 ^a^	0.339 ± 0.010 ^a^	0.323 ± 0.019 ^a^
Proline (Pro)	0.157 ± 0.001 ^a^	0.156 ± 0.004 ^a^	0.143 ± 0.012 ^a^
Methionine (Met)	0.064 ± 0.011 ^a^	0.059 ± 0.011 ^a^	0.051 ± 0.001 ^a^
Serine (Ser)	6.034 ± 0.443 ^a^	5.603 ± 0.753 ^a^	5.587 ± 0.503 ^a^
Glycine (Gly)	0.103 ± 0.014 ^a^	0.099 ± 0.014 ^a^	0.118 ± 0.013 ^a^
Phenylalanine (Phe)	0.638 ± 0.069 ^a^	0.578 ± 0.066 ^a^	0.582 ± 0.037 ^a^
Total	28.006 ± 2.302 ^b^	32.361 ± 1.978 ^a^	28.533 ± 1.556 ^ab^

Note: Each value is the mean ± SE of three independent experiments. Different superscript letters within rows indicate significant differences (*p* < 0.05).

**Table 2:** Key VOCs with rOAV > 1 in different treatments.

CAS	Name	Type	Odor	rOAV		
				CK	C8	C16
65767-22-8	(5Z)-Octa-1,5-dien-3-one	Ketone	Geranium, metallic	100.00	99.69	79.24
14901-07-6	β-Ionone	Terpenoids	Floral, woody, sweet, fruity	51.50	100.00	100.00
4312-99-6	1-Octen-3-one	Ketone	Mushroom	-	20.82	16.57
79-77-6	trans-β-Ionone	Terpenoids	Floral, woody	1.80	3.50	3.50
50746-10-6	3-Mercapto-3-methylbutylformate	Ester	Sulfury, onion	-	2.15	1.14
66-25-1	Hexanal	Aldehyde	Green	<1	1.57	1.03
557-48-2	(E,Z)-2,6-Nonadienal	Aldehyde	Cucumber, green	-	1.42	<1
18829-56-6	(E)-2-Nonenal	Aldehyde	Fatty, green, cucumber, fruity	1.65	<1	<1
2463-53-8	2-Nonenal	Aldehyde	Fatty, green, cucumber, fruity	1.32	<1	<1
5870-68-8	Ethyl 3-methylpentanoate	Ester	Fruity	1.30	<1	<1
140-26-1	Phenethyl isovalerate	Ester	Floral, fruity, sweet	1.19	<1	<1
3658-80-8	Dimethyl trisulfide	Sulfur compounds	Sulfury, onion	1.20	<1	<1

## Data Availability

The original contributions presented in this study are included in the article and Supplementary Materials. Further inquiries can be directed to the corresponding author.

## Supplementary Materials

The following supporting information can be downloaded at: https://www.mdpi.com/article/10.3390/foods14223914/s1, List of all identified volatile compounds detected by GC-MS in green-ripe and red-ripe cherry tomato fruits.
